# Development of the Crossmatch Test in Kidney Transplantation Up to the Virtual Level

**DOI:** 10.3390/jcm14041288

**Published:** 2025-02-15

**Authors:** Nataša Katalinić, Tajana Crnić Marčetić, Zlatko Trobonjača, Franco Barin-Turica, Sanja Balen

**Affiliations:** 1 Tissue Typing Laboratory, Clinical Institute for Transfusion Medicine, Clinical Hospital Center Rijeka, 51000 Rijeka, Croatia; tajanacrnic@gmail.com (T.C.M.); sanja.balen@medri.uniri.hr (S.B.); 2 Department of Clinical Laboratory Diagnostics, Faculty of Medicine in Rijeka, University of Rijeka, 51000 Rijeka, Croatia; 3 Department of Physiology, Immunology and Pathophysiology, Faculty of Medicine, University of Rijeka, 51000 Rijeka, Croatia; zlatko.trobonjaca@uniri.hr; 4 Independent Researcher, 51000 Rijeka, Croatia; francobarint@gmail.com

**Keywords:** complement-dependent cytotoxicity test, crossmatch, histocompatibility testings, kidney transplantation, single-antigen bead, virtual crossmatch

## Abstract

The Human Leukocyte Antigen (HLA) system forms the central part of the immune system and is crucial in the recognition and elimination of “non-self” antigens. While this role of the HLA system is essential in the effective defense of the organism against pathogens, it is undesirable in organ and tissue transplantation because it enables the recognition of mismatched HLA molecules of the donor as being foreign and stimulates the graft rejection reaction. Organ transplantation involves the introduction of antigens that are more or less mismatched to the recipient; therefore, in order to achieve the best possible match in the HLA system between the recipient and the donor, a whole series of immunogenetic tests is performed, including crossmatching (XM). If performed before kidney transplantation, it represents the final in vitro test to rule out the presence of donor-specific antibodies, which may cause graft rejection and which may not have been detected by earlier serum screening. The beginning of XM was marked by the complement-dependent cytotoxicity (CDC) method developed by Terasaki and colleagues in 1964. Later, as a result of advances in technology and the need for methods that overcome the limitations of CDC, flow cytometry and Luminex XM assays were developed. The introduction of solid-phase technology brought a new dimension to the detection of low-level HLA antibodies and the determination of their specificities, which enabled the development and implementation of the virtual XM test (vXM). It is an *in silico* test that assesses the immunological match between the recipient and the organ donor based on the analysis of the specificity of the antibodies present in the recipient’s serum and the HLA typing of the organ donor. Each method has its own advantages and limitations, which are described below and need to be taken into account, considering their significant impact on clinical application in kidney transplantation.

## 1. Introduction

The Human Leukocyte Antigen (HLA) system plays a central role in the immune system. It is crucial in recognizing “self” versus “non-self” antigens, enabling the stimulation and regulation of the immune response directed toward “non-self” molecules while simultaneously maintaining tolerance to “self” antigens. Although this function of the HLA system is necessary for the efficient defense of the organism against pathogens, it is undesirable in organ and tissue transplantation.

The HLA complex is organized into three regions (class I, II and III) located on the short arm of chromosome 6. HLA Class I and II genes encode proteins whose primary role is to bind peptide antigens and present them to the immune system for recognition by T lymphocytes [1]. Class III genes are located between the HLA class I and class II regions and do not include HLA genes but contain genes encoding complement components and some other factors. HLA genes are closely linked and are inherited as an HLA haplotype according to classical Mendelian genetics. Class I antigens are expressed on the surface of almost all nucleated cells. They are responsible for presenting intracellular antigenic peptides to cytotoxic, CD 8+ T lymphocytes. Class II antigens are present on antigen-presenting cells and are involved in presenting extracellular molecules to helper, CD 4+ T lymphocytes.

The HLA system is the most intensively studied genetic region in humans. As more than 40,000 alleles have been discovered since the identification of the first HLA antigen in 1958, the need to create a uniform nomenclature arose early on. Therefore, in 1968, the World Health Organization Committee on the Nomenclature of HLA Factors was established, which is responsible for standardizing, updating, and maintaining the terminology and nomenclature associated with the HLA system [2].

The clinical role of the HLA system is evident in many areas, such as susceptibility to autoimmune diseases, defense against infectious diseases, cancer immunotherapy, paternity testing, drug safety, and has a particularly critical aspect in solid organ and stem cell transplantation, where it represents the main histocompatibility barrier [1,2].

## 2. Methods

This systematic review followed PRISMA (Preferred Reporting Items for Systematic Reviews and Meta-Analyses) guidelines. We performed a literature search for relevant articles in PubMed (MEDLINE) and Web of Science databases up to 1 November 2024 using a combination of Medical Subject Headings (MeSH) terms and text words: “Kidney Transplantation Crossmatch”, “Renal Transplantation Crossmatch”, “Complement Dependent Cytotoxicity Crossmatch“, “Flow Cytometry Crossmatch“, “Luminex Crossmatch”, “Virtual Crossmatch”, and “HLA” in various combinations with the Boolean operators -and, -or. We identified eligible studies using the following inclusion criteria: (1) written in English; (2) studies comparing at least two crossmatch methods; (3) studies reporting sensitivity and specificity for XM methods; (4) studies including clinical outcomes (graft survival, rejection rates); and (5) randomized controlled trials (RCTs), cohort studies, and case–control studies. The exclusion criteria were: (1) articles written in a language other than English (2) case reports, editorials, reviews without original data, articles presented as abstracts in a congress or seminar; (3) studies lacking a clear methodology for crossmatch testing; (4) results on non-human population. The selection process comprised initially the evaluation of titles and abstracts of all electronic records. Subsequently, articles were selected when obtained in full-text form, excluding studies failing to report outcomes of interest. Two authors (N.K./T.C.M.) individually reviewed the database search results assessing the article eligibility utilizing a standardized Excel document to extract relevant data from the included articles. Data extracted included information on study design and sample size, crossmatch method(s) evaluated, performance metrics (sensitivity, specificity), clinical outcomes (graft survival, rejection rates), and economic evaluation (cost per test, resource utilization).

### Search Strategy Results

**Figure 1:**
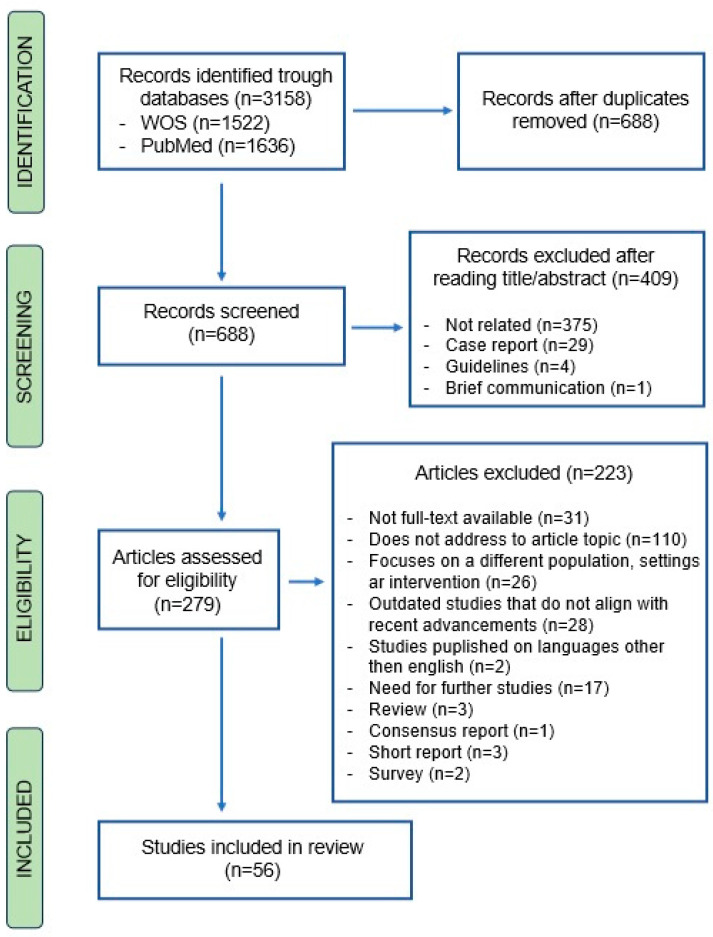
PRISMA flowchart.

The initial search resulted in 3158 studies. After removing 2470 duplicates using the reference manager (Mendeley), 688 articles were selected for further screening, resulting in 279 studies for additional assessing for the eligibility. Finally, 56 studies were selected for review after applying the inclusion and exclusion criteria. The study selection flow chart is shown in Figure 1.

## 3. Immunogenetic Testing of the Recipient and Donor Before Kidney Transplantation

Organ transplantation involves the introduction of a large amount of antigens that are, to a greater or lesser extent, foreign to the recipient. In order to achieve the best possible HLA compatibility between recipients and donors and to reduce the incentive for the development of an immune graft rejection reaction, a series of immunogenetic tests are performed prior to the organ transplantation. These tests include identification of HLA alleles/antigens polymorphism (tissue typing), serum screening for the presence of HLA antibodies and determination of their specificity, as well as autologous and allogeneic crossmatch tests [3].

HLA typing is defined as the identification of HLA class I and II antigens and genes polymorphism through serological and molecular tests. It is performed for both the recipient and the organ donor to determine compatibility in the HLA system. Historically, the first was serological typing, which was enabled by the discovery of HLA antibodies in the sera of pregnant women and multitransfused patients [4,5,6]. With this approach, the basic technique is complement-mediated microlymphocytotoxicity. Although it is a simple and inexpensive method, due to its limitations (viable lymphocytes are required, ability to detect rare or weak antigens is limited, etc.) this method has mostly been abandoned, and molecular typing techniques based on PCR (Polymerase Chain Reaction) technology such as sequence-specific oligonucleotide (SSO) probe hybridization, sequence-specific primers (SSP), real time (RT) PCR or DNA sequencing are now widely used [7].

The development of tissue typing methods has enabled the achievement of a high level of resolution, that is, the ability to distinguish different alleles within the HLA gene family, which has consequently led to a better understanding of antibody recognition patterns and progress in HLA matching from the antigen to the epitope level [8].

Serum screening is a procedure that tests the presence of anti-HLA antibodies and determines their specificity if they are present in the patient. These antibodies target HLA molecules expressed on the cell surface that are highly polymorphic and can vary greatly between individuals, allowing for the development of antibodies with a wide spectrum of specificities.

In the context of kidney transplantation, anti-HLA antibodies can be pre-existing (or previously formed) antibodies and de novo antibodies [9]. Pre-existing antibodies are present in the recipient before transplantation as a result of previous exposure to mismatched HLA antigens through blood transfusion, pregnancy, or a previous transplant. These can increase the risk of hyperacute or acute rejection of the transplanted organ. De novo antibodies develop after transplantation as a result of exposure to mismatched HLA molecules expressed in the transplanted organ. They contribute to chronic graft rejection.

A comprehensive definition of the anti-HLA antibody profile and its monitoring in the patient’s serum allows for the identification of antigens that are considered “unacceptable” for transplantation, since their presence on the donor organ can cause antibody-mediated graft rejection [10]. Therefore, in patients on the waiting list for kidney transplantation, serum is periodically screened for the presence of anti-HLA antibodies. Currently, two screening methods are most commonly used: cell-based tests (CDC method), where results are expressed as a percentage of panel-reactive antibodies (%PRA), and solid-phase techniques (Luminex method), which have enabled the introduction of calculated PRA (cPRA) or virtual PRA (vPRA). vPRA is based on the HLA specificities of HLA that are considered unacceptable for a sensitized transplant recipient and is calculated based on the frequency of antigens among organ donors in the studied population. Thus, vPRA represents the percentage of donors expected to have unacceptable HLA antigens to which the potential kidney recipient is sensitized [11,12].

## 4. Crossmatch Test (XM)

The presence of antibodies to mismatched HLA antigens of the organ donor triggers an immune rejection response in the recipient and is considered one of the most significant obstacles to successful transplantation. Therefore, before kidney transplantation, a final in vitro crossmatch test is performed to verify whether donor-specific antibodies (DSAs) are present in the recipient’s serum that may not have been previously detected in screening and that could cause graft rejection.

The first recorded serological crossmatch test was related to blood transfusion and was performed in 1908 [13]. In organ transplantation, the development of the crossmatch test is closely linked to research and discoveries about the HLA system. The first successful kidney transplant was performed between identical twins in 1954, four years before the discovery of the HLA system. At that time, the “crossmatch” was performed by transplanting a 2.5 × 2.5 cm skin graft between the twins. A control, autologous graft was placed 1 cm above the allogeneic one. After a little more than a month, a biopsy of the transplanted skin was performed. Macroscopic and microscopic differences between autologous and allogeneic grafts were negligible, which was considered to be evidence of tissue compatibility between twins [14]. The discovery of the first HLA antigens marked the beginning of the era of determining tissue compatibility between organ recipients and donors. The importance of the humoral immune response in transplantation was highlighted by Terasaki and Patel in the late 1960s. They found that immediate kidney graft loss occurred in 24 out of the 30 (80%) transplantations performed across positive XM results demonstrating that the presence of cytotoxic antibodies in the recipient directed against HLA antigens expressed on donor kidney cells (DSAs) could lead to the development of antibody-mediated immune and hyperacute graft rejection [15]. This discovery led to the development of the CDC crossmatch test and its mandatory use before transplantation to assess the immunological risk of transplanted organ rejection.

The same authors in their seminal work (1969) also observed that not all kidneys transplanted with positive XM were rejected, while the graft failed immediately in 8 of 195 (4%) kidneys transplanted with negative XM. Even then, they discussed the reasons for false positive and false negative results in XM tests, pointing to several factors such as technical problems, the presence of nonspecific antibodies, immune responses that cannot be detected at the time of testing, and limitations in the sensitivity of the test methods themselves [15].

Therefore, in addition to the described allogeneic XM, during the immunogenetic testing of a potential kidney recipient, an autologous XM must also be performed in which the serum and cells (HLA antigens) belong to the same person. Autoantibodies are thought not to be harmful to the graft, but their presence may cause false positive XM results before transplantation and unnecessary delay of transplantation. Therefore, it is essential to investigate the presence and characteristics of autoantibodies in patients in a timely manner and distinguish them from potential HLA alloantibodies, which could have a deleterious effect on graft function and survival.

The testing protocol for allogeneic XM varies from center to center, mainly depending on the patient’s HLA sensitization. Several methods are used to perform XM.

### 4.1. Complement-Dependent Cytotoxicity Method (CDC)

The introduction of the crossmatch (XM) test was marked by the complement-dependent cytotoxicity (CDC) method, developed by Terasaki and colleagues in 1964 [16]. This test uses the recipient’s serum and donor lymphocytes, which are isolated from the peripheral blood of a living donor and the lymph nodes and/or spleen of a deceased donor.

**Figure 2:**
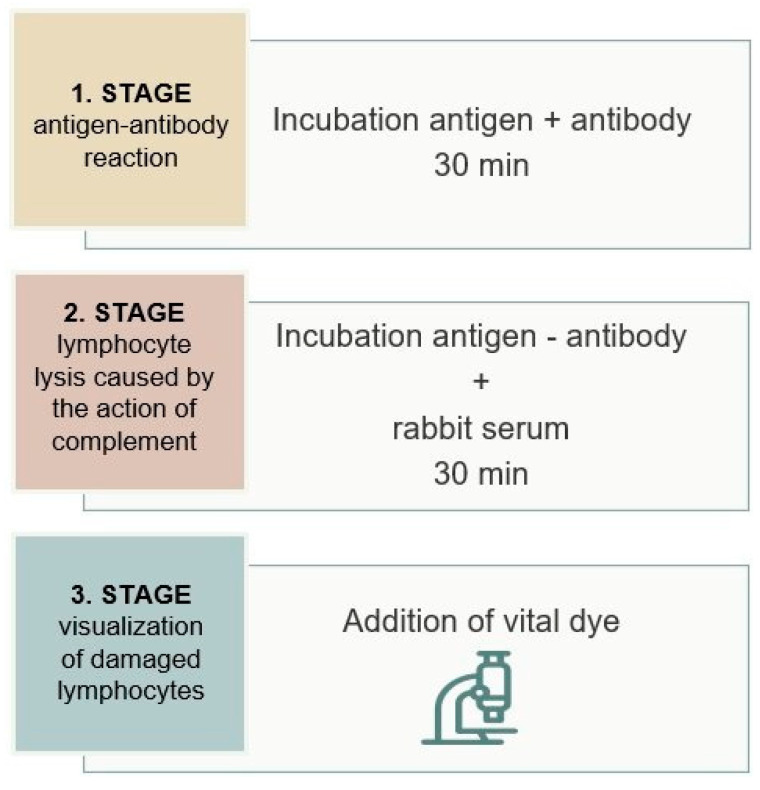
Principle of the CDC test.

As shown in Figure 2, the test is based on a three-step reaction. First, lymphocytes (antigens) are incubated with the recipient’s serum (antibodies), forming an antigen–antibody complex if the patient’s cells contain an antigen specific to the antibody. In the second phase of the reaction, rabbit serum is added as a source of complement. On cells where antigen–antibody complexes are present on the surface, complement is activated, leading to the formation of the Membrane Attack Complex (MAC) that damages the cell membrane. In the third phase of the reaction, vital dyes are added that penetrate the interior of the damaged cells, which, under an inverted microscope, indicate a positive reaction. The intensity of the reaction is scored based on the ratio of viable to dead cells, ranging from 1 (negative) to 8 (100% of damaged cells).

A negative XM result means that there is no reaction between the donor lymphocytes (white blood cells) and the recipient serum. However, it should be taken into account that a negative result does not completely rule out the possibility of DSAs, which may be present at low levels or can be undetectable by CDC-based methods. In the case of a positive XM, DSAs are present in the recipient’s serum. If IgG anti-HLA antibodies are present, they will cause an immune graft rejection reaction, while IgM anti-HLA antibodies are not considered clinically significant in kidney transplantation [17]. To differentiate the antibody class, the XM test is performed with the addition of dithiothreitol (DTT), which breaks the disulfide bonds of IgM pentamers, creating monomers that are not able to activate complement. The DTT concentration is adjusted to affect only the disulfide bonds in the IgM molecule, leaving IgG antibodies intact. A positive crossmatch result without DTT and a negative result with DTT indicates the presence of IgM antibodies, which is not a contraindication for transplantation. A positive crossmatch result with and without DTT indicates the presence of IgG antibodies and requires further evaluation (Table 1).

To improve the CDC XM test, especially due to its low sensitivity, several modifications have been introduced, such as prolonged incubation times, separation of T and B lymphocytes, different incubation temperatures, and the use of anti-human globulin (AHG) alongside the DTT treatment of serum [18].

Advantages of the CDC XM test: High predictive value in identifying clinically significant anti-HLA antibodies that activate complement.

Disadvantages of the CDC XM test: Poor reproducibility, subjective interpretation of results, dependence on the proper viability and number of lymphocytes, and the inability to detect class II anti-HLA antibodies unless T and B lymphocytes are separated, which is a complex and demanding procedure.

False negative results may occur due to the low sensitivity of the test (unable to detect low-titer anti-HLA antibodies) or weak expression of HLA molecules on cell surfaces, which can be affected by medications such as statins or steroids [19].

False positive results can be caused by antibody reactions with non-HLA molecules expressed on cells, the reactivity of autoantibodies, or immune complexes [20].

### 4.2. Flow Cytometry Method

According to research by Patel and Terasaki, about 15% of sensitized recipients rejected the kidney early after transplantation despite a negative CDC XM [15]. As a result, there was a need for more sensitive XM techniques to detect lower levels of DSAs, leading to the development of the flow cytometry crossmatch (FXM) test, first performed before transplantation in 1983 [21]. 

This is a cell-based test in which a suspension of donor lymphocytes (antigens) and recipient’s serum (antibodies) are incubated, allowing eventually present antibodies to bind to the complementary antigen. After washing, a fluorescein-labeled anti-IgG antibody is added, which binds to the antibody in the antigen–antibody complex (DSAs). The fluorescence intensity is detected and measured as the cells pass through laser beams, and the data are processed by computer software. A negative FXM result indicates the absence of donor-specific antibodies detected by the test (as DSAs may be below the detection threshold of the test), while a positive result shows the presence of donor-specific antibodies, regardless of their complement-activating ability. Additionally, using specific monoclonal antibodies allows for the differentiation of T and B lymphocyte subpopulations, which is useful for identifying antibodies against class I HLA molecules (expressed on T and B lymphocytes) or class II HLA molecules (expressed only on B lymphocytes) [18,22].

Some laboratories have developed modified FXM protocols to detect lytic antibodies, reduce processing time (the Halifax and Halifaster protocols), and increase the specificity and sensitivity XM by pronase treatment, donor HLA-specific FXM, etc. [23,24,25,26,27,28,29,30].

Advantages of the FXM test: One of the significant advantages of the flow cytometry method is its higher sensitivity compared to the CDC XM, allowing the detection of low-titer DSAs, detection of immunoglobulins of clinical significance regardless of their complement-activating ability, and objective result interpretation, additionally at lower costs [31].

Disadvantages of the FXM test: False negative or positive results. False negative results can occur due to low HLA molecule expression on donor cells and the presence of non-IgG or non-HLA antibodies that will not be detected; the test does not detect low avidity/affinity DSAs that can be washed out during the washing steps, high background signals in the negative serum control reaction, or an unfavorable antigen–antibody ratio due to an excessive number of cells or small serum volume [32].

False positive results may be caused by insufficient washing after incubating the lymphocyte suspension with antibodies, a low background signal in the negative control reaction, autoantibodies (rarely), or the use of therapeutic antibodies such as anti-thymocyte globulin, rituximab (anti-CD20), alemtuzumab (anti-CD52), basiliximab (anti-CD25), and daclizumab (anti-CD25) [22,33].

### 4.3. Solid-Phase Method (Luminex)

The introduction of Luminex technology in the early 2000s brought a new dimension to the detection of anti-HLA antibodies and the determination of their specificities. Solid-phase techniques enabled the detection of very low-titer anti-HLA antibodies of both class I and II, for which clinical significance has not yet been precisely defined. The Luminex XM (LumXM) test uses color-coded microbeads coated with monoclonal antibodies specific to HLA class I and II, onto which HLA molecules from donor organ cells, obtained by cell lysis, bind during incubation. When the recipient’s serum is added, eventually present DSAs will bind to the donor’s HLA antigens, and this reaction is detected by a secondary anti-human IgG antibody labeled with phycoerythrin (PE). Collected data are analyzed with a Luminex analyzer with xPONENT software and the MatchIt Antibody Analysis program. Previous studies on the clinical role of LumXM test have shown that recipients with positive results for anti-HLA class II antibodies do not have an increased risk factor for graft survival, while a positive LumXM result for anti-HLA class I antibodies is predictive of antibody-mediated graft rejection [34,35,36,37]. In conclusion, LumXM represents a combination of cell-based and solid-phase assays that can only be implemented in pre-transplant risk assessment if used with other XM methods.

Advantages of the LumXM test: Higher sensitivity compared to CDC XM, clearly expressed results for specific HLA antibodies, no need for viable donor lymphocyte cells, and the lysate can be stored for extended periods for valid post-transplant monitoring [37].

Disadvantages of the LumXM test: Compared to Luminex SAB tests, LumXM test shows lower sensitivity, especially for anti-HLA antibodies targeting HLA-A and HLA-B loci with low MFI values in SAB (Single-Antigen Bead) testing. DSA specificities for anti-HLA -Cw, -DQ, and -DP are most often undetectable, as are IgM anti-HLA antibodies. In addition to false negative results, false positives can occur, especially in recipients who had a positive autologous XM.

Given that they improve transplant outcomes, LumXM and SAB tests are cost-effective, and, due to the advantages and disadvantages of both tests, they are often combined [38,39].

### 4.4. Virtual Test

**Figure 3:**
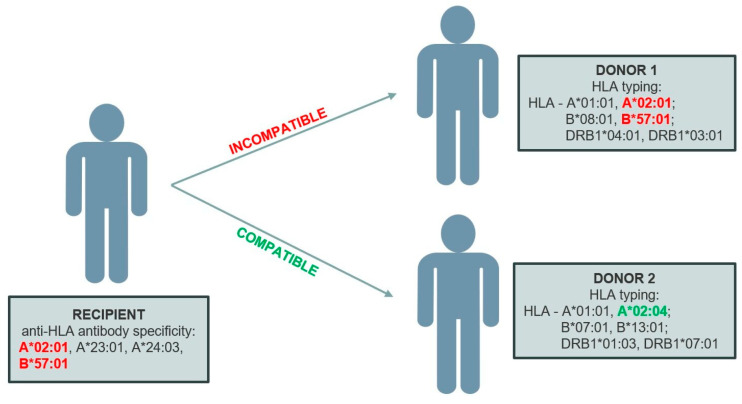
Principle of the vXM.

The virtual crossmatch (vXM) is an in silico test used to assess the immunological compatibility between the organ recipient and donor based on an analysis of the specificity of antibodies present in the recipient’s serum and the HLA typing of the donor organ. This involves comparing the HLA alleles of the donor with unacceptable antigens for the recipient at the allele level. If the donor’s allele (antigen) is found in the recipient’s unacceptable antigen profile, the vXM will be positive (Figure 3).

The widespread application of the vXM was enabled by the development of highly sensitive solid-phase methods used for detecting and determining the specificity of anti-HLA antibodies, as well as the development of molecular HLA typing techniques that allow for high-resolution identification of HLA allele polymorphisms. SAB tests represent the most sensitive method for detecting and determining the specificity of anti-HLA antibodies. On each set of microbeads, purified recombinant HLA glycoproteins (antigens) of only one specificity are conjugated. Since each set of polystyrene microbeads has a unique spectral signature, up to 100 different bead sets can be detected, meaning that the presence of anti-HLA antibodies can be tested for 100 different antigens in a single test. The basic principle of this method is that, after incubating the microbeads with the patient’s serum, any present IgG anti-HLA antibodies specifically bind to antigens conjugated to the beads while unbound antibodies are washed away. Bound anti-HLA antibodies are then labeled with anti-human IgG labeled with a fluorescent dye, phycoerythrin (PE), which is excited by a laser. The Luminex system uses two lasers—a green laser (532 nm) and a red laser (635 nm). The green laser excites the PE dye, emitting a unique signal that identifies bound anti-HLA antibodies. The red laser excites fluorochrome dyes within the beads, allowing for the differentiation of different bead sets within a single test. The test result is expressed as the mean fluorescence intensity (MFI) of the beads [40].

One of the most significant advantages of the vXM is the possibility of rapid assessment of immunological compatibility between the organ recipient and the donor. A negative result allows for immediate transplantation without waiting for physical crossmatch test results. This significantly reduces cold ischemia time, which is critical for the success of cadaveric transplants, increases the number of potential recipients, and decreases waiting time for transplantation. This is particularly important in heart and lung transplantation, where additional time required for performing a physical XM increases the likelihood of organ damage [41,42]. Studies show that patients transplanted using the vXM have similar long-term outcomes compared to those transplanted using prospective physical XM. Additionally, the average number of days on the waiting list for transplantation has significantly decreased since the introduction of the vXM [43].

The results of the vXM have shown high concordance with physical XM results in many studies [44]. One significant study was conducted by Taylor and colleagues, who described their 10-year experience with selectively omitting physical XMs in cadaveric transplants in 2010 [45]. The authors found no difference between patients who had a physical XM before transplantation and those who had only a virtual XM regarding graft function, acute rejection, or graft and recipient survival. Croatia is a member of Eurotransplant, an organ exchange organization in which the vXM is currently used to decide on organ allocation, while many laboratories, including the one in Rijeka, still perform physical XM with and without DTT prior to transplantation [46]. In the Tissue Typing Laboratory Rijeka, a comparison was made between CDC and virtual XM test results performed before kidney transplants from 2018 to 2023. Discrepancy was observed in 2% of crossmatch test results, in which the CDC XMs were positive while the vXM results were negative. Further investigations showed that CDC positivity was mainly caused by non-HLA antibodies and, in a smaller number of cases, by complement-binding IgG autoantibodies that were present due to autoimmune disease [47].

Advantages of the test: This test has the highest sensitivity and specificity compared to other methods, with no interference from antibodies against antigens outside the HLA system. It does not require viable cells but relies on comprehensive HLA typing of the donor and the assessment of the recipient’s antibody profile. It can be completed in a few minutes (less than an hour), which allows for faster decision-making regarding transplantation and increases the likelihood that organs can be transported over long distances without significantly affecting the outcome of the transplant. Additionally, it reduces the use of preliminary XMs with accompanying cost savings [48,49,50].

Disadvantages of the test: Since the vXM is based on the results of SAB tests, all the characteristics of Luminex SAB testing apply to the interpretation of the vXM. Variations in the expression of HLA molecules on actual, native cells are different from the conformational structure and expression level of HLA molecules on microbeads in the SAB test. Therefore, the limitations of the vXM are thoroughly outlined to ensure the correct interpretation of results in clinical applications.

False negative results can be caused by:

- “Peanut butter” effect: This refers to the ability to detect a small amount of antibody targeted to an antigen on one bead; when spread across many beads, the signal can fall below the set threshold, analogous to spreading peanut butter on one slice of bread versus distributing it across all slices of a loaf. Recent studies have not confirmed this effect [51].
- Prozone effect: This occurs when serum components interfere with the detection of anti-HLA antibodies, such as high levels of antibodies that may activate complement, leading to C1 deposition on the beads, the presence of IgM antibodies, immune complexes, intravenous immunoglobulin, thymoglobulin, or other factors that can interfere with secondary antibody binding. Complement-mediated interference in SAB tests can be reduced by treating sera with ethylenediaminetetraacetic acid (EDTA), heat, or DTT before testing [52].
- Antibodies to rare alleles that are not represented on the microbeads of the SAB test.
- Undetected HLA antibodies due to concentrations below the test’s sensitivity threshold, which is determined by the minimum MFI value. In such cases, the clinical significance of the antibody specificity must be considered based on the sensitizing event and previous test results.
- Unreported sensitizing events after the last serum screening.
- Complement-activating antibodies, which are associated with a higher incidence of acute graft rejection reactions, cannot be distinguished from non-complement-activating antibodies using SAB tests or the vXM. For this purpose, adding C1q or C3d components to the SAB test is recommended [53,54].
- False positive results may be caused by:
- Detecting antibodies that are clinically insignificant. Since MFI values are not standardized, the detection of antibodies that are not clinically significant may occur due to test hypersensitivity, leading to misinterpretation. As a result, transplantation may be unnecessarily delayed due to a false positive result or inappropriate immunosuppressive therapy may be applied after the procedure.
- The presence of potentially interfering autoantibodies resulting from autoimmune disease [55]. One of the procedures performed in this case is the autologous XM test, which can distinguish between auto- and alloantibodies.
- The production process during reagent preparation and the binding of HLA molecules to microbeads, which may lead to conformational changes, denaturation, and the exposure of a new epitope (which does not exist on the native molecule) or cryptic antigens (which are otherwise unavailable to antibodies) with which antibodies will react [30,56,57,58,59,60].
- The presence of therapeutic antibodies such as rituximab and anti-thymocyte globulin [61].
- “Natural” HLA antibodies, i.e., HLA antibodies detected in individuals without any known sensitization events, which are currently considered nonspecific. It is believed that they may arise due to cross-reactivity following bacterial or viral infections, such as influenza and hepatitis C, or after vaccination. Environmental factors such as microorganisms, food proteins, and allergens are considered as possible causes. Pro-inflammatory events, such as surgical procedures or trauma, are also associated with an increase in titers and the broadening of the specificity of anti-HLA antibodies [62,63,64,65,66].
- Non-HLA antibodies. Non-HLA antigens are molecules outside the HLA system expressed on lymphocytes. They arise as a product of nonsynonymous single nucleotide polymorphisms (SNPs) that result in a change in the codon, inserting a different amino acid into the polypeptide, creating polymorphic peptides recognized as “non-self” by the immune system. Non-HLA molecule mismatches between the donor and recipient will trigger an immune response and the formation of specific antibodies. Research is underway to investigate the association between the development of graft rejection in kidney and other organ transplants and antibodies targeting non-HLA molecules, such as antibodies against vimentin, endothelin receptors, angiotensin II receptors, and other antigens [67,68].

**Figure 4:**
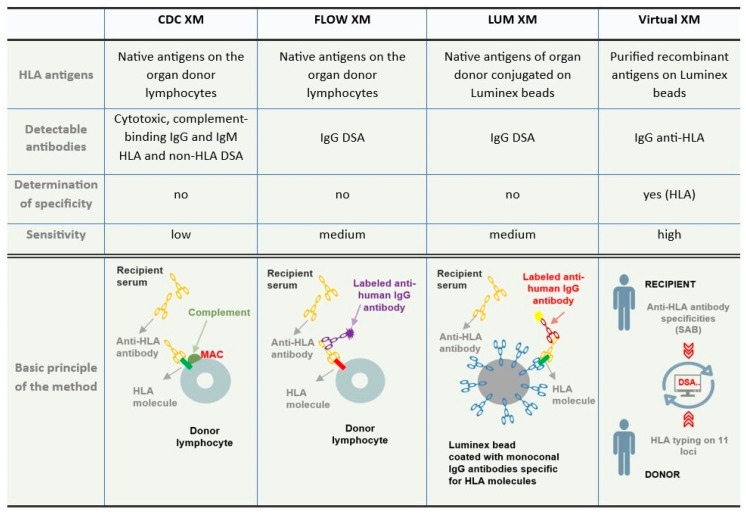
Comparison of the basic features of crossmatch test methods.

A comparison of the basic characteristics of crossmatch test methods is shown in Figure 4.

### 4.5. Clinical Application of Virtual Crossmatch Testing in Kidney Transplantation

A crossmatch test is part of the immunogenetic assessment of patients to determine immune compatibility between the organ recipient and donor in order to estimate immunological risk for the organ recipient. Before transplantation, this procedure determines the presence of DSAs in the patient’s serum, which can adversely affect graft function and survival as well as the patient’s clinical condition.

As early as 1969, Patel and Terasaki demonstrated that, in kidney transplantation, 80% of grafts were rejected after a positive CDC XM [15]. Due to its high predictive value, the CDC-based XM method was considered the gold standard in kidney transplantation for decades. Initially, every positive result (whether from a “fresh” or “historical” serum sample) was considered a contraindication for transplantation. However, research aided by the development of immunosuppressive drugs demonstrated that a positive XM with “historical” serum samples did not significantly affect transplantation outcomes if the XM with a “fresh” serum sample was negative. To overcome the limitations of the CDC XM test (despite modifications), more sensitive tests such as XM methods based on flow cytometry and solid-phase assays were developed [21].

Today, molecular methods allow for HLA allele sequencing, and solid-phase assays enable the detection of low-titer antibodies and precise determination of specificity, driving the advancement of XM methods to the level of virtual testing. In virtual XM, the specificity of anti-HLA antibodies detected in the recipient is compared to the HLA antigen profile of the potential organ donor, making comprehensive and accurate HLA typing of the donor a key requirement for an accurate vXM. However, there is ongoing discussion about actual HLA typing of the second field, which may result in the rejection of some otherwise acceptable offers [69,70,71]. 

One of the greatest challenges of the vXM is determining clinically significant donor-specific antibodies. The test is based on the results of SAB tests expressed as MFI values. The threshold value is not standardized, differing from laboratory to laboratory, and due to the characteristics of Luminex technology, there is a fine line between sensitivity and hypersensitivity. Depending on the test manufacturer, antibodies with an MFI lower than 1000–2000 are generally considered to be of low immunological risk and clinically insignificant. Antibodies with higher MFI values must be taken into account when interpreting the results, keeping in mind several specificities of the test. First, the fact that MFI is a semi-quantitative value should be considered. The assumption that antibodies with higher MFI values are more clinical significant compared to antibodies with lower MFI is not always correct for several reasons. Not all HLA antigens are equally immunogenic. For instance, HLA-A2, -B35, and -B44 antigens are highly immunogenic, so there is a high likelihood of graft rejection if the DSAs of these specificities are present, even at lower MFI values. Additionally, antigens from the HLA-C, -DQ, and -DP loci are more represented on the beads in SAB tests than on native cells. Therefore, the threshold MFI value indicating clinically significant antibodies for these loci should be higher than for others. Furthermore, in patients with previous transplants, some centers exclude their donor’s mismatched antigens, regardless of their presence in the SAB test, due to a memory immune response [72,73].

Understanding the limitations of each test is crucial for accurately interpreting laboratory results. The above XM methods have their own advantages and disadvantages, which must be considered when selecting the most appropriate method for the center’s needs. Factors such as technical characteristics, performance difficulty and result interpretation, financial aspects, and the possibility of clinical and pharmacotherapeutic management of the transplant recipient must also be considered. Additionally, one method does not exclude another, and applicability can be defined based on specific patient groups (e.g., non-sensitized, sensitized, and/or highly sensitized patients). The CDC and flow cytometry methods use viable, native cells, and the reactions in these tests are the closest to those that actually occur in the body. Furthermore, the experience in interpreting and understanding the test results in clinical application makes these methods, particularly CDC, still the method of choice in many transplant centers. On the other hand, solid-phase tests use recombinant molecules, and reactions will not fully reflect biological mechanisms. However, compared to cellular tests, they have high sensitivity and specificity, enabling the detection of DSAs in a low titer; additionally, they have become the basis of the vXM, which enters into increasingly widespread applications. For comparison with other methods, the vXM detects anti-HLA antibodies that have an MFI ≤ 1000–2000 in the SAB test (depending on the test manufacturer). The flow cytometry-based XM test is positive when MFI ≥ 2500; for LXM, the MFI cut-off value is 1000 and the CDC XM is positive for MFI values higher than 5000–10,000 [32,74,75,76,77,78]. These data are useful for correlating results from different methods and interpreting them in a clinical context [78]

Above all, it is important to highlight that the results of anti-HLA antibody serum screening and XM tests are interdependent, meaning that, with an accurate determination of the specificity of anti-HLA antibodies in the patient’s serum, it is possible to predict the result of the XM with high certainty. In addition to XM results, analyzing the results of screening is equally important to gain a better understanding of the patient’s immune status and assess the risks associated with the potential donor.

A challenge and a special approach are required by sensitized, especially highly sensitized, transplant candidates (vPRA ≥ 85% in Eurotransplant).

These groups of patients require a comprehensive approach based on the results of SAB tests, information related to the immune status of the recipient, previous sensitizing events, and the complete HLA typing of the donor. It is necessary to have extensive knowledge of the specificity and characteristics of the antibodies that will be included in the analysis when performing a vXM. The STAR (Sensitization in Transplantation: Assessment of Risk) working group published recommendations in 2022 on the assessment of anti-HLA antibodies and the evaluation of therapies aimed at reducing antibody titers. The report emphasizes the importance of using solid-phase immunoassays (SPIs) and suggests that performing serum dilutions can increase the accuracy of HLA antibody detection, especially in patients with high antibody levels. These tests are recommended because of their sensitivity and specificity in identifying DSAs, which are crucial for assessing immunological risk in transplantation [79]. However, while SAB detection of potential DSAs is very accurate in patients with low sensitization, when the specificity of the antibody is well defined, this may not be true for highly sensitized patients. Therefore, many laboratories continue to rely on the results of the physical XM test in such patients as a valuable contribution to the determination of clinically significant DSAs [80].

Due to the various aspects that need to be taken into account, efforts are ongoing to create guidelines/consensus on the management of sensitized patients awaiting kidney transplant that include immune risk stratification [81,82]. Recently, the ENGAGE II working group classified sensitized candidates into five categories based on SAB and CDC and/or FXM results. They recommend that patients with DSAs and positive CDC XM should not be transplanted since they carry the highest immune risk for alograft rejection, as is generally mostly accepted [83]. In cases where the procedure is considered, desensitization before transplantation must be performed to provide a negative CDC XM. These protocols, alone or in combination, include plasma exchange, intravenous immunoglobulin, immunoadsorption, anti-CD20 monoclonal antibodies, proteasome inhibitors, imlifidase, and other therapeutics, along with other strategies, such as kidney paired donation, prioritization in allocation schemes, etc. The intensive discussion regarding the clinical risk for candidates with pretransplant DSAs with negative physical XM is still ongoing. The conclusion of most studies is that transplantation is not contraindicated in such patients. However, while some authors consider that this group of patients has minimal immunological risk and that desensitization therapy is not necessary, others argue that they carry an increased risk and recommend implementing desensitization strategies before transplantation [84,85,86,87,88,89,90,91,92,93].

Transplant medicine, particularly immunogenetics, is one of the fastest-growing fields in medicine and science. New technologies and methods of molecular typing, the discovery of the 3D structure of HLA molecules, and the development of sensitive techniques to test for the presence of anti-HLA antibodies have led to a better understanding of the complex patterns of humoral immunity. The terms epitope, eplet, and triplet have been known for years, and progress in this area has enabled the development of algorithms for tissue matching at the epitope level (HLA epitope matching) using computer programs such as HLAMatchmaker and Predicted Indirectly ReCognizable HLA Epitopes (PIRCHE) [94,95,96]. One of the necessary prerequisites for the wider clinical application of these algorithms is the adoption of a unified stand on the definition of clinically significant epitopes and matching strategy. It is known that the properties of epitopes are influenced not only by their conformational structure but also by the electrostatic charge, the types and properties of the amino acids surrounding the eplet, etc. Reactivity to a specific eplet does not necessarily mean that all alleles carrying that eplet are considered unacceptable. It is also important to differentiate between the immunogenicity and antigenicity of epitopes at this level. Immunogenicity refers to the ability to induce a humoral and/or cell-mediated immune response, while antigenicity refers to the ability of a molecule to specifically bind to antibodies and/or surface receptors on T lymphocytes [97]. Immunogenic molecules have antigenic properties, but the reverse is not necessarily true. Assessing the immunogenicity of individual epitopes is crucial in preventing graft rejection [98].

It is also necessary to emphasize the growing potential of using artificial intelligence (AI) in all areas of everyday life as well as in medicine. AI is currently in the research phase, but there is a prospect for its implementation, with the vXM becoming the basis for using AI to determine immunological risk for the recipient before transplantation. In any case, it is expected that the future will open new avenues for further research and discoveries in the field of transplant immunogenetics.

Finally, one of the key elements, regardless of the advancement of science and technology, which forms the foundation of a successful transplantation outcome is good, open, and continuous communication between HLA laboratory staff and transplant clinicians. Every transplant center should assess the risk for each patient individually based on the results of all methods and tests used (screening, XM), all sensitizing events, and all clinical data relevant to assessing the patient’s immune status. Only a personalized approach can ensure appropriate patient care and secure a long-term successful outcome.

## Figures and Tables

**Table 1:** Interpretation of the crossmatch test results without dithiothreitol (CDC-DTT) and with dithiothreitol (CDC + DTT).

CDC-DTT	CDC+DTT	Abbreviation	Interpretation of DSA Presence	Conclusion
Negative	Negative	CDC-DTT (-) CDC+DTT (-)	IgG/IgM negative	XM negative
Positive	Negative	CDC-DTT (+) CDC+DTT (-)	IgG positive IgM negative	XM negative
Positive	Positive	CDC-DTT (+) CDC+DTT (+)	IgG positive	XM positive

## Data Availability

Data sharing is not applicable to this manuscript as no new data were created nor analyzed in this study.

## References

[1] Mosaad Y.M. (2015). Clinical Role of Human Leukocyte Antigen in Health and Disease. Scand. J. Immunol..

[2] Choo S.Y. (2007). The HLA system: Genetics, immunology, clinical testing, and clinical implications. Yonsei Med. J..

[3] Tinckam K.J. (2011). Basic Histocompatibility Testing Methods. Core Concepts in Renal Transplantation.

[4] Dausset J. (1958). Iso-leuco-anticorps [Iso-leuko-antibodies]. Acta Haematol..

[5] van Rood J., Eernisse J.G., van Leeuwen A. (1958). Leucocyte antibodies in sera from pregnant women. Nature.

[6] Payne R., Rolfs M.R. (1958). Fetomaternal leukocyte incompatibility. J. Clin. Investig..

[7] Choi H., Choi E.J., Kim H.J., Baek I.C., Won A., Park S.J., Kim T.G., Chung Y.J. (2024). A walk through the development of human leukocyte antigen typing: From serologic techniques to next-generation sequencing. Clin. Transplant. Res..

[8] Geo J.A., Ameen R., Al Shemmari S., Thomas J. (2024). Advancements in HLA Typing Techniques and Their Impact on Transplantation Medicine. Med. Princ. Pract..

[9] Alelign T., Ahmed M.M., Bobosha K., Tadesse Y., Howe R., Petros B. (2018). Kidney Transplantation: The Challenge of Human Leukocyte Antigen and Its Therapeutic Strategies. J. Immunol. Res..

[10] Süsal C., Roelen D.L., Fischer G., Campos E.F., Gerbase-DeLima M., Hönger G., Schaub S., Lachmann N., Martorell J., Claas F. (2013). Algorithms for the determination of unacceptable HLA antigen mismatches in kidney transplant recipients. Tissue Antigens.

[11] Huber L., Lachmann N., Niemann M., Naik M., Liefeldt L., Glander P., Schmidt D., Halleck F., Waiser J., Brakemeier S. (2015). Pretransplant virtual PRA and long-term outcomes of kidney transplant recipients. Transpl. Int..

[12] Süsal C., Morath C. (2015). Virtual PRA replaces traditional PRA: Small change but significantly more justice for sensitized patients. Transpl. Int..

[13] Oberman H.A. (1981). The crossmatch. A brief historical perspective. Transfusion.

[14] Ellis H. (2015). The first identical twin renal transplant. J. Perioper. Pract..

[15] Patel R., Terasaki P.I. (1969). Significance of the positive crossmatch test in kidney transplantation. N. Engl. J. Med..

[16] Terasaki P.I., McClelland J.D. (1964). Microdroplet assay of human serum cytotoxins. Nature.

[17] McCalmon R.T., Tardif G.N., Sheehan M.A., Fitting K., Kortz W., Kam I. (1997). IgM antibodies in renal transplantation. Clin. Transplant..

[18] Jaramillo A., Ramon D.S., Stoll S.T. (2018). Technical aspects of crossmatching in transplantation. Clin. Lab. Med..

[19] Hönger G., Krähenbühl N., Dimeloe S., Stern M., Schaub S., Hess C. (2015). Inter-individual differences in HLA expression can impact the CDC crossmatch. Tissue Antigens.

[20] Badders J.L., Jones J.A., Jeresano M.E., Schillinger K.P., Jackson A.M. (2015). Variable HLA expression on deceased donor lymphocytes: Not all crossmatches are created equal. Hum. Immunol..

[21] Garovoy M.R., Rheinschmilt M.A., Bigos M., Perkins H., Colombe B., Feduska N., Salvatierra O. (1983). Flow cytometry analysis: A high technology crossmatch technique facilitation transplantation. Transplant. Proc..

[22] Maguire O., Tario J.D., Shanahan T.C., Wallace P.K., Minderman H. (2014). Flow cytometry and solid organ transplantation: A perfect match. Immunol. Investig..

[23] Zieliński M., Zielińska H., Moszkowska G., Dȩbska-Ślizień A., Rutkowski B., Trzonkowski P. (2013). Modified flow cytometry crossmatch detecting alloantibody-related cytotoxicity as a way to distinguish lytic antibodies from harmless in allosensitised kidney recipients. Transplant. Proc..

[24] Liwski R.S., Greenshields A.L., Conrad D.M., Murphey C., Bray R.A., Neumann J., Gebel H.M. (2018). Rapid optimized flow cytometric crossmatch (FCXM) assays: The Halifax and Halifaster protocols. Hum. Immunol..

[25] Vaidya S., Cooper T.Y., Avandsalehi J., Barnes T., Brooks K., Hymel P., Noor M., Sellers R., Thomas A., Stewart D. (2001). Improved flow cytometric detection of HLA alloantibodies using pronase—Potential implications in renal transplantation. Transplantation.

[26] Bearden C.M., Agarwal A., Book B.K., Sidner R.A., Gebel H.M., Bray R.A., Pescovitz M.D. (2004). Pronase treatment facilitates alloantibody flow cytometric and cytotoxic crossmatching in the presence of rituximab. Hum. Immunol..

[27] Brown N.K., Meade J.R., Wang J., Marino S.R. (2017). Reanalysis of the role of pronase treatment of B cells in the flow cytometric crossmatch assay: Fc receptor is not the primary target. Hum. Immunol..

[28] Chen G., Lin L., Tyan D.B. (2020). DSA-FXM: Accelerated Donor-specific Flow Crossmatch Discriminating Class I and II Antibody Specifically and Only to Donor HLA for Determining True Incompatibility. Transplantation.

[29] Ta M., Scornik J.C. (2002). Improved flow cytometric detection of donor-specific HLA class II antibodies by heat inactivation. Transplantation.

[30] Visentin J., Guidicelli G., Bachelet T., Jacquelinet C., Audry B., Nong T., Dubois V., Moreau J.F., Lee J.H., Couzi L. (2014). Denatured class I human leukocyte antigen antibodies in sensitized kidney recipients: Prevalence, relevance, and impact on organ allocation. Transplantation.

[31] Pelletier R.P., Orosz C.G., Adams P.W., Bumgardner G.L., Davies E.A., Elkhammas E.A., Henry M.L., Ferguson R.M. (1997). Clinical and economic impact of flow cytometry crossmatching in primary cadaveric kidney and simultaneous pancreas-kidney transplant recipients. Transplantation.

[32] Rocha Y., Jaramillo A., Neumann J., Hacke K., Palou E., Torres J. (2023). Crossmatch assays in transplantation: Physical or virtual?: A review. Medicine.

[33] Guillaume N. (2018). Improved flow cytometry crossmatching in kidney transplantation. HLA.

[34] Eng H.S., Bennett G., Tsiopelas E., Lake M., Humphreys I., Chang S.H., Coates P.T., Russ G.R. (2008). Anti-HLA donor-specific antibodies detected in positive B-cell crossmatches by Luminex predict late graft loss. Am. J. Transplant..

[35] Billen E.V., Voorter C.E., Christiaans M.H., van den Berg-Loonen E.M. (2008). Luminex donor-specific crossmatches. Tissue Antigens.

[36] Guillaume N., Mazouz H., Piot V., Presne C., Westeel P.F. (2013). Correlation between Luminex donor-specific crossmatches and levels of donor-specific antibodies in pretransplantation screening. Tissue Antigens.

[37] Ameur R.F., Berkani L.M., Belaid B., Habchi K., Saidani M., Djidjik R. (2023). Luminex Crossmatch in kidney transplantation. Scand. J. Immunol..

[38] Chowdhry M., Makroo R.N., Thakur Y., Sharma V., Singh M., Kumar M. (2018). The good, the bad, and the ugly of luminex donor-specific crossmatch. HLA.

[39] Nguyen H.T., Lim W.H., Craig J.C., Chapman J.R., Lord S.J., Howard K., Wong G. (2015). The relative benefits and costs of solid phase bead technology to detect preformed donor specific antihuman leukocyte antigen antibodies in determining suitability for kidney transplantation. Transplantation.

[40] Lachmann N., Todorova K., Schulze H., Schönemann C. (2013). Luminex(^®^) and its applications for solid organ transplantation, hematopoietic stem cell transplantation, and transfusion. Transfus. Med. Hemother..

[41] Bohmig G.A., Fidler S., Christiansen F.T., Fischer G., Ferrari P. (2013). Transnational validation of the Australian algorithm for virtual crossmatch allocation in kidney paired donation. Hum. Immunol..

[42] Zangwill S., Ellis T., Stendahl G., Zahn A., Berger S., Tweddell J. (2007). Practical application of the virtual crossmatch. Pediatr. Transplant..

[43] Yanagida R., Czer L.S., Reinsmoen N.L., Cao K., Rafiei M., De Robertis M.A., Mirocha J., Kass R.M., Kobashigawa J.A., Trento A. (2011). Impact of virtual cross match on waiting times for heart transplantation. Ann. Thorac. Surg..

[44] Baxter-Lowe L.A., Cecka M., Kamoun M., Sinacore J., Melcher M.L. (2014). Center-defined unacceptable HLA antigens facilitate transplants for sensitized patients in a multi-center kidney exchange program. Am. J. Transplant..

[45] Taylor C.J., Kosmoliaptsis V., Sharples L.D., Prezzi D., Morgan C.H., Key T., Chaudhry A.N., Amin I., Clatworthy M.R., Butler A.J. (2010). Ten-year experience of selective omission of the pretransplant crossmatch test in deceased donor kidney transplantation. Transplantation.

[46] Heidt S., Kramer C.S.M., Haasnoot G.W., Schmidt A.H., Zoet Y.M., Claas F.H.J., Vogelaar S. (2024). Introduction of the donor centre virtual crossmatch in Eurotransplant. HLA.

[47] Barin-Turica F. (2024). Virtual Crossmatch Test in Kidney Transplantation. Bachelor’s Thesis.

[48] Bingaman A.W., Murphey C.L., Palma-Vargas J., Wright F. (2008). A virtual crossmatch protocol significantly increases access of highly sensitized patients to deceased donor kidney transplantation. Transplantation.

[49] Tafulo S., Osório E., Mendes C., Liwski R. (2024). Complement-dependent cytotoxicity crossmatch in solid organ transplantation: The gold standard or golden history?. Hum. Immunol..

[50] Olszowska-Zaremba N., Zagożdżon R., Gozdowska J. (2022). Accuracy of virtual crossmatch (VXM) prediction of physical crossmatch (PXM) results of donor specific antibody (DSA) in routine pretransplant settings-a single-center experience. Transpl. Immunol..

[51] Claisse G., Devriese M., Lion J., Maillard N., Caillat-Zucman S., Mooney N., Taupin J.L. (2022). Relevance of Anti-HLA Antibody Strength Underestimation in Single Antigen Bead Assay for Shared Eplets. Transplantation.

[52] Schnaidt M., Weinstock C., Jurisic M., Schmid-Horch B., Ender A., Wernet D. (2011). HLA antibody specification using single-antigen beads—A technical solution for the prozone effect. Transplantation.

[53] Crespo M., Torio A., Mas V., Redondo D., Pérez-Sáez M.J., Mir M., Faura A., Guerra R., Montes-Ares O., Checa M.D. (2013). Clinical relevance of pretransplant anti-HLA donor-specific antibodies: Does C1q-fixation matter?. Transpl. Immunol..

[54] Juhl D., Marget M., Hallensleben M., Görg S., Ziemann M. (2017). Assignment of C1q-binding HLA antibodies as unacceptable HLA antigens avoids positive CDC-crossmatches prior to transplantation of deceased donor organs. Transpl. Immunol..

[55] Schlaf G., Pollok-Kopp B., Schabel E., Altermann W. (2013). Artificially Positive Crossmatches Not Leading to the Refusal of Kidney Donations due to the Usage of Adequate Diagnostic Tools. Case. Rep. Transplant..

[56] Amico P., Hönger G., Mayr M., Schaub S. (2008). Detection of HLA-antibodies prior to renal transplantation: Prospects and limitations of new assays. Swiss. Med. Wkly..

[57] Gutiérrez-Larrañaga M., Riesco L., Guiral S., Irure J., Rodrigo E., Ocejo-Vinyals J., Martorell J., Caro J.L., López-Hoyos M., San Segundo D. (2021). Detection of antibodies to denatured human leucocyte antigen molecules by single antigen Luminex. HLA.

[58] Jucaud V., Ravindranath M.H., Terasaki P.I. (2017). Conformational Variants of the Individual HLA-I Antigens on Luminex Single Antigen Beads Used in Monitoring HLA Antibodies: Problems and Solutions. Transplantation.

[59] Kim B., Kim S., Park Y., Kim H.S. (2021). False-positive reactivity of anti-human leukocyte antigen antibodies detected using the single-antigen bead assay. Hum. Immunol..

[60] Zoet Y.M., Brand-Schaaf S.H., Roelen D.L., Mulder A., Claas F.H., Doxiadis I.I. (2011). Challenging the golden standard in defining donor-specific antibodies: Does the solid phase assay meet the expectations?. Tissue Antigens.

[61] Milongo D., Vieu G., Blavy S., Del Bello A., Sallusto F., Rostaing L., Kamar N., Congy-Jolivet N. (2015). Interference of therapeutic antibodies used in desensitization protocols on lymphocytotoxicity crossmatch results. Transpl. Immunol..

[62] Morales-Buenrostro L.E., Terasaki P.I., Marino-Vázquez L.A., Lee J.H., El-Awar N., Alberú J. (2008). “Natural” human leukocyte antigen antibodies found in nonalloimmunized healthy males. Transplantation.

[63] Hirata A.A., McIntire F.C., Terasaki P.I., Mittal K.K. (1973). Cross reactions between human transplantation antigens and bacterial lipopolysaccharides. Transplantation.

[64] Katerinis I., Hadaya K., Duquesnoy R., Ferrari-Lacraz S., Meier S., van Delden C., Martin P.Y., Siegrist C.A., Villard J. (2011). De novo anti-HLA antibody after pandemic H1N1 and seasonal influenza immunization in kidney transplant recipients. Am. J. Transplant..

[65] El Aggan H.A., Sidkey F., El Gezery D.A., Ghoneim E. (2004). Circulating anti-HLA antibodies in patients with chronic hepatitis C: Relation to disease activity. Egypt. J. Immunol..

[66] Zachary A.A., Lucas D.P., Detrick B., Leffell M.S. (2009). Naturally occurring interference in Luminex assays for HLA-specific antibodies: Characteristics and resolution. Hum. Immunol..

[67] Zhang Q., Reed E.F. (2016). The importance of non-HLA antibodies in transplantation. Nat. Rev. Nephrol..

[68] Helanterä I., Markkinen S., Partanen J., Hyvärinen K. (2024). Novel Aspects of Immunogenetics and Post-Transplant Events in Kidney Transplantation. Transpl. Int..

[69] Cruz T.D., Dames C., Pagaduan L., Cho Y., Kong D., Rajalingam R. (2022). Concurrent use of two independent methods prevents erroneous HLA typing of deceased organ donors—An important strategy for patient safety and accurate virtual crossmatching for broader sharing. Hum. Immunol..

[70] Heidt S., Haasnoot G.W., van der Linden-van Oevelen M.J.H., Claas F.H.J. (2021). Highly Sensitized Patients Are Well Served by Receiving a Compatible Organ Offer Based on Acceptable Mismatches. Front. Immunol..

[71] Bachelet T., Martinez C., Del Bello A., Couzi L., Kejji S., Guidicelli G., Lepreux S., Visentin J., Congy-Jolivet N., Rostaing L. (2016). Deleterious Impact of Donor-Specific Anti-HLA Antibodies Toward HLA-Cw and HLA-DP in Kidney Transplantation. Transplantation.

[72] Baştürk B., Kantaroğlu B., Kavuzlu M., Sarıtürk Ç. (2016). The Most Common HLA Alleles and Anti-HLA Antibodies to Know for Virtual Cross-Match. Exp. Clin. Transplant..

[73] Ellis T.M., Schiller J.J., Roza A.M., Cronin D.C., Shames B.D., Johnson C.P. (2012). Diagnostic accuracy of solid phase HLA antibody assays for prediction of crossmatch strength. Hum. Immunol..

[74] Flynn P.A., Fernando S., Worthington J.E., Poulton K.V. (2024). Predicting flow cytometry crossmatch results from single-antigen bead testing. Int. J. Immunogenet..

[75] Baranwal A.K., Bhat D.K., Goswami S., Agarwal S.K., Kaur G., Kaur J., Mehra N. (2017). Comparative analysis of Luminex-based donor-specific antibody mean fluorescence intensity values with complement-dependent cytotoxicity & flow crossmatch results in live donor renal transplantation. Indian J. Med. Res..

[76] Pandey P., Pande A., Mandal S., Marik A., Devra A.K., Sinha V.K., Bhatt A.P., Gajway S.Y., Singh R.K., Mishra S. (2023). Detection of donor-specific HLA antibodies: A retrospective observation in 350 renal transplant cases. Transpl. Immunol..

[77] Peräsaari J.P., Jaatinen T., Merenmies J. (2018). Donor-specific HLA antibodies in predicting crossmatch outcome: Comparison of three different laboratory techniques. Transpl. Immunol..

[78] Wrenn S.M., Marroquin C.E., Hain D.S., Harm S.K., Pineda J.A., Hammond P.B., Shah D.H., Hillyard S.E., Fung M.K. (2018). Improving the performance of virtual crossmatch results by correlating with nationally-performed physical crossmatches: Obtaining additional value from proficiency testing activities. Hum. Immunol..

[79] Tambur A.R., Bestard O., Campbell P., Chong A.S., Barrio M.C., Ford M.L., Gebel H.M., Heidt S., Hickey M., Jackson A. (2023). Sensitization in transplantation: Assessment of Risk 2022 Working Group Meeting Report. Am. J. Transplant..

[80] Jani V., Ingulli E., Mekeel K., Morris G.P. (2017). Root cause analysis of limitations of virtual crossmatch for kidney allocation to highly-sensitized patients. Hum. Immunol..

[81] Zecher D., Bach C., Preiss A., Staudner C., Utpatel K., Evert M., Jung B., Bergler T., Böger C.A., Spriewald B.M. (2018). Analysis of Luminex-based Algorithms to Define Unacceptable HLA Antibodies in CDC-crossmatch Negative Kidney Transplant Recipients. Transplantation.

[82] Furian L., Bestard O., Budde K., Cozzi E., Diekmann F., Mamode N., Naesens M., Pengel L.H.M., Schwartz Sorensen S., Vistoli F. (2024). European Consensus on the Management of Sensitized Kidney Transplant Recipients: A Delphi Study. Transpl. Int..

[83] Arreola-Guerra J.M., Castelán N., de Santiago A., Arvizu A., Gonzalez-Tableros N., López M., Salcedo I., Vilatobá M., Granados J., Morales-Buenrostro L.E. (2016). C1Q Assay Results in Complement-Dependent Cytotoxicity Crossmatch Negative Renal Transplant Candidates with Donor-Specific Antibodies: High Specificity but Low Sensitivity When Predicting Flow Crossmatch. J. Transplant..

[84] Lee H., Lee H., Sun I.O., Park J.H., Park J.W., Ban T.H., Yang J., Kim M.S., Yang C.W., Chung B.H. (2024). Pre-transplant crossmatch-negative donor-specific anti-HLA antibody predicts acute antibody-mediated rejection but not long-term outcomes in kidney transplantation: An analysis of the Korean Organ Transplantation Registry. Front. Immunol..

[85] Senev A., Lerut E., Van Sandt V., Coemans M., Callemeyn J., Sprangers B., Kuypers D., Emonds M.P., Naesens M. (2019). Specificity, strength, and evolution of pretransplant donor-specific HLA antibodies determine outcome after kidney transplantation. Am. J. Transplant..

[86] Süsal C., Ovens J., Mahmoud K., Döhler B., Scherer S., Ruhenstroth A., Tran T.H., Heinold A., Opelz G. (2011). No association of kidney graft loss with human leukocyte antigen antibodies detected exclusively by sensitive Luminex single-antigen testing: A Collaborative Transplant Study report. Transplantation.

[87] Tian J., Li D., Alberghini T.V., Rewinski M., Guo N., Bow L.M. (2015). Pre-transplant low level HLA antibody shows a composite poor outcome in long-term outcome of renal transplant recipients. Ren. Fail..

[88] Adebiyi O.O., Gralla J., Klem P., Freed B., Davis S., Wiseman A.C., Cooper J.E. (2016). Clinical Significance of Pretransplant Donor-Specific Antibodies in the Setting of Negative Cell-Based Flow Cytometry Crossmatching in Kidney Transplant Recipients. Am. J. Transplant..

[89] Amrouche L., Aubert O., Suberbielle C., Rabant M., Van Huyen J.D., Martinez F., Sberro-Soussan R., Scemla A., Tinel C., Snanoudj R. (2017). Long-term Outcomes of Kidney Transplantation in Patients with High Levels of Preformed DSA: The Necker High-Risk Transplant Program. Transplantation.

[90] Couzi L., Araujo C., Guidicelli G., Bachelet T., Moreau K., Morel D., Robert G., Wallerand H., Moreau J.F., Taupin J.L. (2011). Interpretation of positive flow cytometric crossmatch in the era of the single-antigen bead assay. Transplantation.

[91] David-Neto E., Souza P.S., Panajotopoulos N., Rodrigues H., Ventura C.G., David D.S., Lemos F.B., Agena F., Nahas W.C., Kalil J.E. (2012). The impact of pretransplant donor-specific antibodies on graft outcome in renal transplantation: A six-year follow-up study. Clinics.

[92] Hönger G., Wahrmann M., Amico P., Hopfer H., Böhmig G.A., Schaub S. (2010). C4d-fixing capability of low-level donor-specific HLA antibodies is not predictive for early antibody-mediated rejection. Transplantation.

[93] Kwon H., Kim Y.H., Kim J.Y., Choi J.Y., Shin S., Jung J.H., Park S.K., Han D.J. (2019). The results of HLA-incompatible kidney transplantation according to pre-transplant crossmatch tests: Donor-specific antibody as a prominent predictor of acute rejection. Clin. Transplant..

[94] Tambur A.R. (2018). HLA-Epitope Matching or Eplet Risk Stratification: The Devil Is in the Details. Front. Immunol..

[95] Goodman R.S., Taylor C.J., O’Rourke C.M., Lynch A., Bradley J.A., Key T. (2006). Utility of HLAMatchmaker and single-antigen HLA-antibody detection beads for identification of acceptable mismatches in highly sensitized patients awaiting kidney transplantation. Transplantation.

[96] Zeevi A., Girnita A., Duquesnoy R. (2006). HLA antibody analysis: Sensitivity, specificity, and clinical significance in solid organ transplantation. Immunol. Res..

[97] Zhang J., Tao A. (2015). Antigenicity, Immunogenicity, Allergenicity. Allergy Bioinform..

[98] Bezstarosti S., Kramer C.S.M., Claas F.H.J., de Fijter J.W., Reinders M.E.J., Heidt S. (2022). Implementation of molecular matching in transplantation requires further characterization of both immunogenicity and antigenicity of individual HLA epitopes. Hum. Immunol..

